# T72. VERBAL MEMORY AND VOXEL BASED MORPHOMETRY IN FIRST EPISODE NON-AFFECTIVE PSYCHOSIS: A PROCESS ORIENTED APPROACH

**DOI:** 10.1093/schbul/sby016.348

**Published:** 2018-04-01

**Authors:** Rosa Ayesa-Arriola, Esther Setien-Suero, Diana Tordesillas-Gutierrez, Benedicto Crespo-Facorro

**Affiliations:** 1 Marqués de Valdecilla University Hospital, IDIVAL, University of Cantabria; 2 Marques de Valdecilla University Hospital, IDIVAL, University of Cantabria, CIBERSAM

## Abstract

**Background:**

Deficits in auditory-verbal memory have been reported by the vast majority of published research in schizophrenia and also detected in first episode psychosis (FEP), confirming they are already present at the early stages of the illness. However, the specific neurocognitive constructs underlying defective verbal memory and their neuroanatomical correlates remains poorly understood in schizophrenia spectrum disorders patients.

**Methods:**

Data on the Rey Auditory Verbal Learning Test (RAVLT), a widely used verbal memory measure that provides a range of performance indexes to evaluate distinct memory processes including: a) acquisition/learning b) sensitivity to interference c) retrieval; d) retention or rate of forgetting; e) and retrieval efficiency was available for 388 FEP patients and 184 healthy controls (HC). In 218 FEP patients and 146 HC, structural magnetic resonance imaging data were analysised using Voxel based morphometry (VBM) toolbox.

**Results:**

The FEP group showed significantly lower results on acquisition/learning, delayed recall as well as higher rates of forgetting. They also exhibited a significant sensitivity to retroactive but not proactive interference. We also found significant correlations between bilateral frontal lobe morphometry and proactive interference as well as between right frontal lobe morphometry and retroactive interference. Rate of forgetting was significantly correlated with right occipital cortex morphometry. Those with higher rates of forgetting and proactive and retroactive interference demonstrated further gray matter reductions in frontal and occipital cortical areas.

**Discussion:**

The application of a process oriented approach to the neuropsychological evaluation of verbal memory allows a finer-grained analysis of the neurocognitive constructs underlying defective verbal memory in FEP. The results suggest specific relationships between different neuroanatomical structures and discrete memory processes with these structures playing an important role in verbal memory deficits found in FEP.

